# Characterization of laser cooling in microgravity via long-term operations in TianGong-2 space lab

**DOI:** 10.1093/nsr/nwac180

**Published:** 2022-08-29

**Authors:** De-Sheng Lü, Wei Ren, Yuan Sun, Tang Li, Qiu-Zhi Qu, Bin Wang, Lin Li, Jian-Bo Zhao, Xin Zhao, Jing-Wei Ji, Mei-Feng Ye, Jing-Feng Xiang, Wei-Biao Chen, Yu-Zhu Wang, Liang Liu

**Affiliations:** CAS Key Laboratory of Quantum Optics and Center of Cold Atom Physics, Shanghai Institute of Optics and Fine Mechanics, Chinese Academy of Sciences, Shanghai 201800, China; Aerospace Laser Engineering Department, Shanghai Institute of Optics and Fine Mechanics, Chinese Academy of Sciences, Shanghai 201800, China; CAS Key Laboratory of Quantum Optics and Center of Cold Atom Physics, Shanghai Institute of Optics and Fine Mechanics, Chinese Academy of Sciences, Shanghai 201800, China; Aerospace Laser Engineering Department, Shanghai Institute of Optics and Fine Mechanics, Chinese Academy of Sciences, Shanghai 201800, China; CAS Key Laboratory of Quantum Optics and Center of Cold Atom Physics, Shanghai Institute of Optics and Fine Mechanics, Chinese Academy of Sciences, Shanghai 201800, China; CAS Key Laboratory of Quantum Optics and Center of Cold Atom Physics, Shanghai Institute of Optics and Fine Mechanics, Chinese Academy of Sciences, Shanghai 201800, China; Aerospace Laser Engineering Department, Shanghai Institute of Optics and Fine Mechanics, Chinese Academy of Sciences, Shanghai 201800, China; CAS Key Laboratory of Quantum Optics and Center of Cold Atom Physics, Shanghai Institute of Optics and Fine Mechanics, Chinese Academy of Sciences, Shanghai 201800, China; Aerospace Laser Engineering Department, Shanghai Institute of Optics and Fine Mechanics, Chinese Academy of Sciences, Shanghai 201800, China; CAS Key Laboratory of Quantum Optics and Center of Cold Atom Physics, Shanghai Institute of Optics and Fine Mechanics, Chinese Academy of Sciences, Shanghai 201800, China; Aerospace Laser Engineering Department, Shanghai Institute of Optics and Fine Mechanics, Chinese Academy of Sciences, Shanghai 201800, China; CAS Key Laboratory of Quantum Optics and Center of Cold Atom Physics, Shanghai Institute of Optics and Fine Mechanics, Chinese Academy of Sciences, Shanghai 201800, China; Aerospace Laser Engineering Department, Shanghai Institute of Optics and Fine Mechanics, Chinese Academy of Sciences, Shanghai 201800, China; CAS Key Laboratory of Quantum Optics and Center of Cold Atom Physics, Shanghai Institute of Optics and Fine Mechanics, Chinese Academy of Sciences, Shanghai 201800, China; Aerospace Laser Engineering Department, Shanghai Institute of Optics and Fine Mechanics, Chinese Academy of Sciences, Shanghai 201800, China; CAS Key Laboratory of Quantum Optics and Center of Cold Atom Physics, Shanghai Institute of Optics and Fine Mechanics, Chinese Academy of Sciences, Shanghai 201800, China; Aerospace Laser Engineering Department, Shanghai Institute of Optics and Fine Mechanics, Chinese Academy of Sciences, Shanghai 201800, China; CAS Key Laboratory of Quantum Optics and Center of Cold Atom Physics, Shanghai Institute of Optics and Fine Mechanics, Chinese Academy of Sciences, Shanghai 201800, China; Aerospace Laser Engineering Department, Shanghai Institute of Optics and Fine Mechanics, Chinese Academy of Sciences, Shanghai 201800, China; CAS Key Laboratory of Quantum Optics and Center of Cold Atom Physics, Shanghai Institute of Optics and Fine Mechanics, Chinese Academy of Sciences, Shanghai 201800, China; Aerospace Laser Engineering Department, Shanghai Institute of Optics and Fine Mechanics, Chinese Academy of Sciences, Shanghai 201800, China; CAS Key Laboratory of Quantum Optics and Center of Cold Atom Physics, Shanghai Institute of Optics and Fine Mechanics, Chinese Academy of Sciences, Shanghai 201800, China; Aerospace Laser Engineering Department, Shanghai Institute of Optics and Fine Mechanics, Chinese Academy of Sciences, Shanghai 201800, China; Aerospace Laser Engineering Department, Shanghai Institute of Optics and Fine Mechanics, Chinese Academy of Sciences, Shanghai 201800, China; CAS Key Laboratory of Quantum Optics and Center of Cold Atom Physics, Shanghai Institute of Optics and Fine Mechanics, Chinese Academy of Sciences, Shanghai 201800, China; CAS Key Laboratory of Quantum Optics and Center of Cold Atom Physics, Shanghai Institute of Optics and Fine Mechanics, Chinese Academy of Sciences, Shanghai 201800, China; Aerospace Laser Engineering Department, Shanghai Institute of Optics and Fine Mechanics, Chinese Academy of Sciences, Shanghai 201800, China

**Keywords:** laser cooling, polarization-gradient cooling, orbital microgravity, space lab

## Abstract

The invention of laser cooling has fundamentally influenced the research frontier of atomic physics and quantum physics, and recently an intense focus has been on the studies of cold atom physics in microgravity environments. Herein, we report the results of our laser cooling experiment in TianGong-2 space lab, which operated for 34 consecutive months in orbit. Over such an extended operation time, the quality of laser cooling did not experience any significant decline, while the properties of laser cooling in orbital microgravity were systematically studied. In particular, we demonstrate magneto-optical trapping and polarization-gradient cooling in orbit and carefully examine their performances. A comparison of the in-orbit and on-ground results indicates that a higher cooling efficiency exists in microgravity, including a smaller loss rate during the trapping and cooling process and lower ultimate temperature of laser-cooled atoms. Our progress has laid the technical foundations for future applications of cold atoms in space missions with operation times of the order of years.

## Teaser

Observation of highly efficient laser cooling in microgravity through conducting cold atom experiment in a space lab with successful and consistent performance for almost three years since 2016.

## INTRODUCTION

Since the invention of laser cooling and trapping of atoms [1–6], cold atom physics and its applications have rapidly developed. Typically, the temperature of laser-cooled atoms can reach as low as a few microkelvin, corresponding to several photon recoils. With sub-recoil cooling techniques such as evaporative or sideband cooling, the cold atom ensemble can arrive at the exotic states of a Bose–Einstein condensate (BEC) [7,8] or degenerate Fermi gas [9–11], which are the standard tools of quantum simulation with neutral atoms [12–14]. Cold atoms have become an essential platform in metrology, quantum sensing, quantum information and precision measurement of fundamental physical constants, such as in microwave atomic clocks [15], optical atomic clocks [16–18], cold atom interferometers [19–24], atomic qubit platform [25–27] and measurement of the electron’s electric dipole moment [28,29]. These experiments are generally operated at ground level where the influence of gravity is inevitable and persistent.

However, the study of cold atom physics in microgravity has recently attracted significant interest. BECs were realized in drop tower and sounding rocket experiments with microgravity times of several seconds and several minutes, respectively [30,31], which can serve as ultracold atom sources for future space missions [32]. A cold atom interferometer operating in microgravity was also demonstrated in a parabolic flight [33]. In particular, the observation of a BEC in the International Space Station has been reported [34], and intense on-going efforts have been devoted to sub-nanokelvin physics in the space lab [35]. Moreover, the cold atom platform in space is believed to have great potential for applications for quantum sensing and fundamental physics, and several promising proposals have been suggested, including the detection of gravitational waves and search for dark matter [36,37]. These proposals typically require long-term in-orbit experiments running continuously for years. Therefore, a systematic study of laser cooling in orbital microgravity with an extended operation time is essential for future developments in this area.

On September 15, 2016, our cold atom clock assembly, under the cold atom clock experiment in space (CACES) mission, was launched into space with the TianGong-2 (TG-2) space lab. It worked properly without the need for external maintenance until the deorbit of TG-2. We have extensively investigated the properties of laser cooling in this space lab, in addition to its applications in cold atom microwave clocks [38,39]. In particular, magneto-optical trapping (MOT) and polarization-gradient cooling (PGC) of ^87^Rb atoms were routinely conducted in orbit for 34 consecutive months with reliable and consistent performance. In this article, we present and analyze the results of such a long-term laser cooling experiment in orbital microgravity. We compare the in-orbit and on-ground results, from which we digest the essential features and underlying physics mechanisms of our experimental observations. Based on our findings, we discuss advantages and challenges of cold atom projects in space missions.

## RESULTS

### Design of the experiment

**Figure 1.**
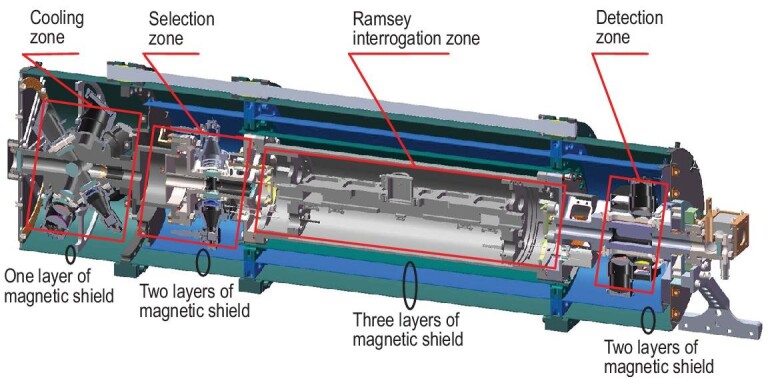
Schematic drawing of the physical package. In particular, it contains a three-layer magnetic shield around the Ramsey interrogation zone and a two-layer magnetic shield around the detection zone.

Our experimental apparatus was carefully designed and constructed to satisfy the TG-2 space lab’s strict requirements of weight, volume, power consumption and safety [38–41]. During long-term operation, the performance of laser cooling and trapping was stable and consistent for nearly three years until TG-2 space lab’s mission concluded. The basic layout of this physical package is illustrated in Fig. 1. The physical package maintains an ultrahigh vacuum (UHV) environment. It consists of four individual functional zones connected by flanges, and its vacuum is supported by two 3 L/s ion pumps. Delta seals and indium were applied to all the flanges, while the optical windows were sealed with Viton^®^ O-rings and indium. Furthermore, to prevent the downgrade of the vacuum level in the Ramsey interrogation zone that occupies a significant length, two groups of getters were placed on the internal sides around the two ends of the vacuum chamber, and each group had four getters. The physical package can maintain UHV for days, even if the ion pumps stop functioning. Its vacuum level when operated in orbit was better than 1 × 10^−7^ Pa.

**Figure 2.**
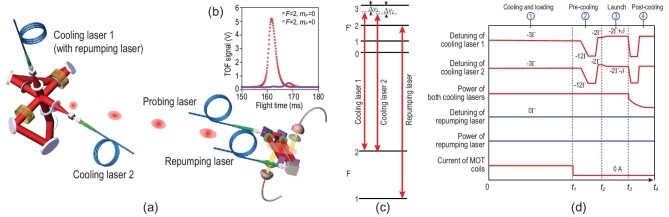
Basic setup of the laser cooling experiment in orbit. (a) Compact folded optical path. The repumping laser is mixed into one of the cooling beams. (b) TOF signal. (c) Energy level corresponding to the cooling laser and repumping laser used in the MOT. (d) Timing sequence. One typical experiment cycle contains four stages that are cooling and loading, pre-cooling, launch and post-cooling. The blue lines represent information about the repumping laser while the red lines are for the cooling lasers and the current of MOT coils. In the post-cooling stage, the PGC lasers are turned off in an adiabatic way.

The setup for the laser cooling experiment is shown in Fig. 2(a). The key part is a specially designed compact magneto-optical trap (CMOT), in which two independent laser beams follow folded optical paths to form a six-beam three-dimensional cooling configuration. The implementation of a folded optical path not only effectively reduces the mass and complexity, but also improves the mechanical robustness. As shown in Fig. 2(c), the cooling lasers are red detuned to the *F* = 2 ↔ *F*′ = 3 transition and the repumping laser is tuned to the resonance with the *F* = 1 ↔ *F*′ = 2 transition of the ^87^Rb D2 line. The frequency and power of both laser beams are precisely controlled by five acousto-optic modulators [41]. The atoms are cooled and loaded in the CMOT from the background vapor, and then the captured cold atoms are further cooled for several milliseconds by the PGC, after the magnetic gradient field is turned off. Subsequently, they are launched by moving optical molasses, which are formed by changing the relative frequency difference between the two cooling lasers. The cold atoms are eventually cooled by the PGC, before they leave the cooling laser beams. The probing process takes place 75 cm downstream. The detailed timing sequence is shown in Fig. 2(d).

In the detection zone, the signals of cold atoms were obtained via laser excited atomic fluorescence. The induced fluorescence was collected by two 10 × 10 mm^2^ photodiodes with an efficiency of 2% and the photocurrent was converted into a voltage signal by two FET amplifiers. This detection system has a noise level of less than 3 μV/$\sqrt{\text{Hz}}$ over frequencies from 1 to 100 Hz, which can be safely neglected in this experiment. A typical signal obtained via time-of-flight (TOF) measurements in the detection zone is shown in Fig. 2(b). The number of cold atoms arriving at the detection zone can be calculated by the fluorescence of atoms at CMOT or integrating the TOF signal. We calibrated the number of cold atoms in the CMOT by measuring both the fluorescence at the CMOT and TOF signal. We carefully maintained the same TOF conditions for both the in-orbit and on-ground experiments, and observed that the loss of atoms was the same during the flight in both cases.

### Performance of laser cooling in space

**Figure 3.**
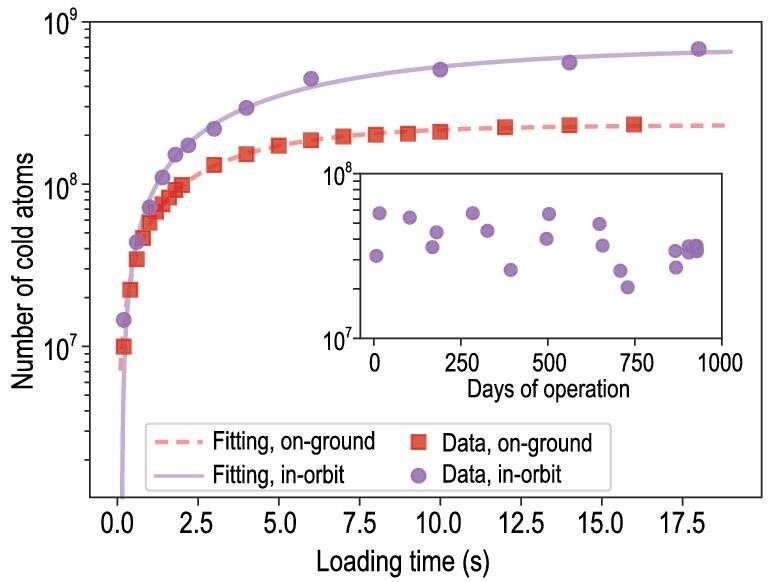
Measurements of cold atom numbers. The main graph shows the number of loaded cold atoms in the MOT versus the loading time, extracted from TOF measurements; squares represent the results on ground and circles represent the results in orbit. The inset shows the number of loaded cold atoms in orbit over an approximately 30-month time span, with the loading time uniformly set at 1 s for these tests. The number drops a little because we intentionally turned down the cooling laser power in later experiments.

The number of cold atoms with respect to different loading times is measured and the result is presented in Fig. 3, with data from before and after the launch of the TG-2 space lab, using the same apparatus and parameter settings. For relatively long loading times in which saturation occurs, the number of cold atoms in orbit is almost three times greater than that on ground. Interpreting the observed enhancements is important for in-orbit laser cooling and trapping. We note that previous space-borne BEC experiments suggested that more condensed atoms were obtained in microgravity than on ground [31,34]. More specifically, the loading process can be simply described by *dN*/*dt* = −η*N* + α, where η, α represent the loss and loading rates, respectively. According to fittings, we find that the loading rate in orbit is 1.5 times higher than that on ground, whereas the loss rate is 0.5 times smaller. Qualitatively, it can be partly understood that the effective trapping potential on the ground deviates from the ideal condition because of the distortion caused by gravity. Moreover, the cooled atoms are constantly pulled downward by gravity such that more atoms accumulate at the lower part of the MOT [42]. The increased density can result in a higher collision rate, possibly leading to a greater overall loss rate. For the in-orbit operation over 30 months, the CMOT had been running repeatedly numerous times with consistent and reliable performance; the numbers of captured cold atoms during the long-term observation are also shown in Fig. 3.

In orbital microgravity, the trapping potential along one pair of counter-propagating cooling laser beams can be described by the spring constant κ of the MOT, and the zero potential point coincides with the zero position of the magnetic gradient field. Under the presence of gravity, the MOT’s force on an atom of velocity $\vec{v}$ and position $\vec{r}$ is given by $\vec{F} = -\beta \vec{v} - \kappa \vec{r} - mg\vec{e}_z$ and the trapping potential changes to *V* = κ*r*^2^/2 + *mgz*, where β is the damping coefficient, *m* is the atomic mass and $\vec{e}_z$ is the unit vector along the *z* axis (aligned with the gravity). Subsequently, the zero potential point is displaced by *z*_0_ = −2*mg*/κ, and the maximum trapping potential is no longer uniform along the three pairs of cooling laser beams. The trapping potential on ground is not ideal, and it is anticipated that a higher atom loss rate and a lower accumulated atom number will occur in comparison with the microgravity environment. Another possible contribution to the increase in in-orbit cold atom numbers is from the atoms in state |*F* = 2, *m_F_* = 0〉 during the atom-laser interaction, which experience gravity as a dominant force on ground. A related effect has been discussed in [34].

**Figure 4.**
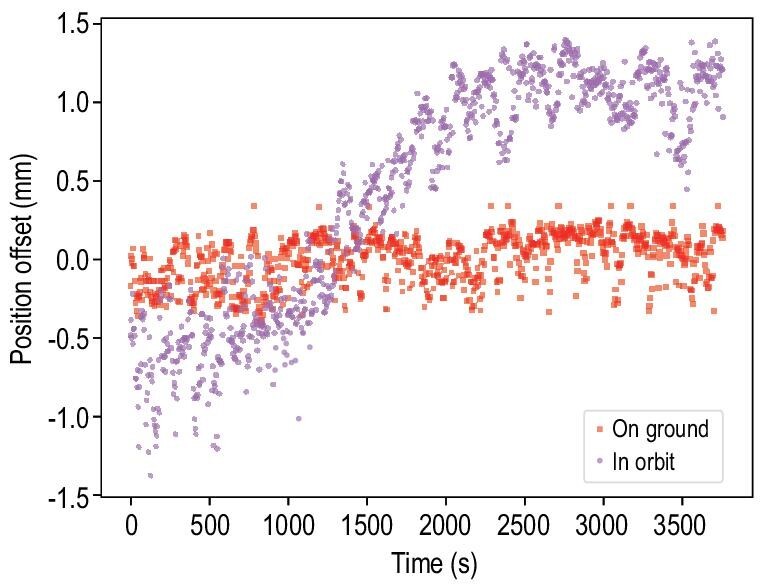
Central positions of the cold atom cloud along the longitudinal direction. Here the zero point of the vertical axis represents the center of the CMOT structure.

Monitoring the center position of the cold atom cloud in the MOT is also a key part of characterization. The center position is deduced based on the TOF measurements. More specifically, while the positions of photodiodes are fixed, the velocity of the launched cold atoms is determined by setting the frequency difference of the moving optical molasses laser beams, and the arrival time can be precisely extracted from the TOF signal. Figure 4 shows a typical result of measuring the cold atom cloud center positions along the longitudinal axis. As expected, the center position remained typically unchanged during the ground tests, whereas it underwent notable changes during the flight in orbit. We observed that it was also affected by changes in the terrestrial magnetic field. Therefore, more attention and effort are required for magnetic shielding in the future for in-orbit laser cooling and trapping experiments, especially if they are intended for tasks such as precision measurements.

### Temperature of cold atoms in space

**Figure 5.**
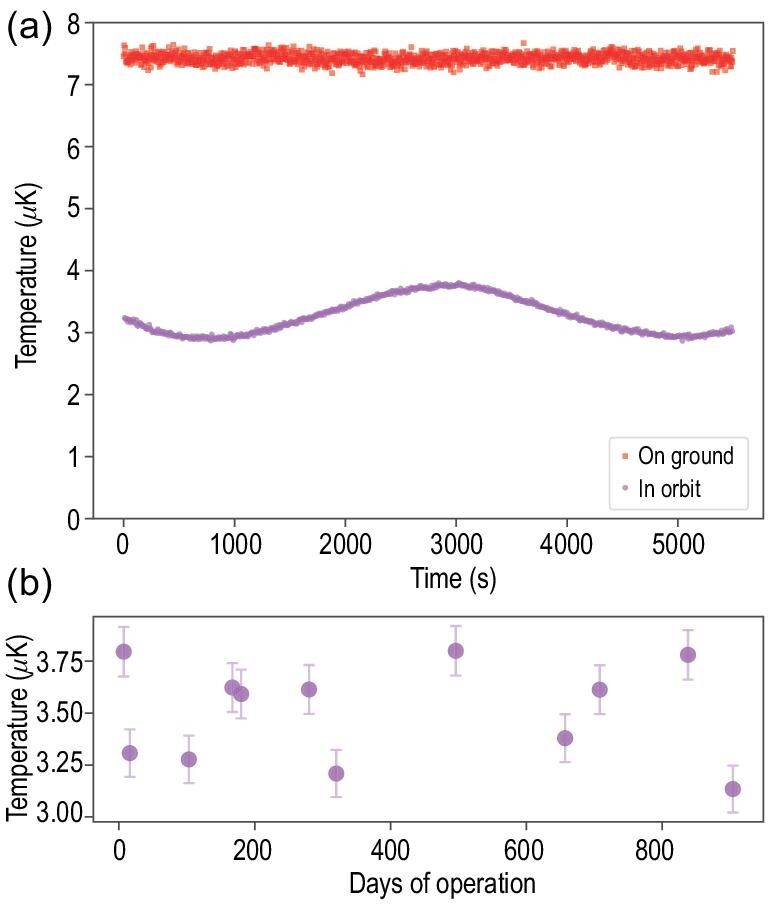
Measurements of the cold atom temperature. (a) Comparison between the results of the cold atom temperature measurement on ground and in orbit. In both cases, the temperature is much lower than the Doppler limit, as a consequence of PGC. (b) Temperature of cold atoms in orbit over an approximately 30-month time span.

The cold atom temperature of the PGC process in the post-cooling stage can be deduced from the TOF signals. We compare the on-ground and in-orbit temperatures using the same experimental parameters, as shown in Fig. 5(a). It contains the results of multiple consecutive launches of cold atoms, with a total time span of approximately 90 minutes, corresponding to the time period of TG-2 moving around the Earth for one cycle. The temperature of cold atoms on ground reaches 7.4 ± 0.3 μK, and this value stays reasonably stable. In orbital microgravity, however, the temperature drops to 3.3 ± 0.5 μK, significantly lower than the on-ground value in the same experimental setting. As discussed earlier, this observation demonstrates the influence of gravity on the PGC process and indicates that the microgravity environment is an attractive and suitable place for studying cold atom physics [35]. Further experiments indicated that the temperature remained reasonably consistent during long-term operation, as shown in Fig. 5(b). Moreover, regular variations occur in the in-orbit temperature measurements, and we deduce that such variations are mostly caused by the change in the terrestrial magnetic field owing to the motion of the space lab, which corroborates the influence of the magnetic field on the PGC process in theory. The CMOT area has only one layer of magnetic shield, which does not provide a sufficient shield rate with respect to the subtle requirements of the PGC process.

Previous theoretical considerations of laser cooling often did not emphasize the influence of gravity. Only recently, experiments on cold atoms in microgravity have been conducted and the role of gravity in laser cooling and trapping processes has begun to draw more attention. In typical idealized Doppler cooling processes, the velocities of cold atoms eventually center at zero, and the cooling force profile is symmetric with respect to the velocity directions. In the presence of gravity, the atom needs to maintain a non-zero velocity, in order to generate a net non-zero laser force to balance gravity. In other words, gravity constantly accelerates the atom until it reaches the equilibrium speed *v_g_*. For a two-level atom in the one-dimensional case, when the laser force can be described as a scattering force, this kinematics condition can be expressed as


$$\begin{eqnarray*}
mg&=&\frac{1}{2}\hbar ks_0\gamma \bigg \lbrace \bigg [1+s_0+ \bigg (\frac{2(\delta +kv_g)}{\gamma }\bigg )^2 \bigg ]^{-1}\\
&& - \bigg [1+s_0+ \bigg (\frac{2(\delta -kv_g)}{\gamma }\bigg )^2 \bigg ]^{-1} \bigg \rbrace .
\end{eqnarray*}$$

We note that such a calculation is a simplification of the actual process. The details of the cooling transition are much more complicated than the model of two-level atom’s Doppler cooling, in which an extra repumping laser is employed together with the cooling laser. Substituting a typical set of parameters for our experiment, *s*_0_ = 4, γ = 2π × 6.07 MHz and δ = −2π × 42.0 MHz, yields the result *v_g_* = 3.6 cm/s, which is approximately 0.01γ/*k*. Such velocity values are already comparable to the effective velocity range of the PGC [3,5,6]. Therefore, from the theoretical point of view, the PGC process becomes responsible for offsetting the gravity in the on-ground experiments described by such a set of parameters.

To obtain a lower temperature, the PGC of the post-cooling stage is usually carried out with cooling lasers of a relatively large detuning and small intensity, which reduces the scattering rate of atoms for suppressing heating. This typically leads to a PGC force profile with an effective range of less than 0.01γ/*k* and a maximum acceleration of the order of a few tens of m/s^2^, where gravity can have a significant impact. Consequently, compared with the ideal case without the influence of gravity, the effective velocity range of the PGC cooling force is reduced on one side and the damping coefficient is virtually smaller around the zero force point. In other words, for typical laser cooling experiments with similar settings and parameters as discussed above, where Doppler cooling and PGC mechanisms coexist, the outcome temperature increases non-trivially in the presence of gravity. If we take the detailed level structure of the ^87^Rb D2 line into consideration, the experimental data can be interpreted using numerical simulations based on a straightforward physical model. In fact, the influence of gravity on the PGC process as well as other various delicate laser cooling techniques is an interesting topic for future studies in this area, especially with the powerful tool of comparisons between in-orbit and on-ground experiments.

## CONCLUSION

In conclusion, we have successfully conducted the long-term laser cooling experiment in a space lab with a specially engineered physical system. We believe that our in-orbit experimental results over 30 months indicate that microgravity provides an advantageous environment for laser cooling, and standalone cold atom experiments can be performed continuously in a space lab with satisfactory and robust performance for periods of the order of years. This will encourage future cold atom physics projects in space labs. For example, BEC can reach an extremely low temperature that is not accessible on ground, and a cold atom interferometer can have much longer interrogation time with better precision. We are also in the process of designing specially tailored atomic physics tools [43] for these projects. We anticipate that our results will be helpful in many important new directions based on long-term cold atom platforms in orbit, especially in the research areas of quantum sensing and fundamental physics.

## DATA AVAILABILITY

Data underlying the results presented in this paper are not publicly available at this time but may be obtained from the authors upon reasonable request.

## Supplementary Material

nwac180_Supplemental_File
