# Supplementary material for: Characterization of laser cooling in microgravity via long-term operations in TianGong-2 space lab
Source: Natl Sci Rev. 2022 Aug 29;10(4):nwac180. doi: 10.1093/nsr/nwac180 (PMC10115171; doi:10.1093/nsr/nwac180)
Supplement: nwac180_Supplemental_File [file nwac180_supplemental_file.pdf]

Supplemental Material: Characterization of Laser Cooling in Microgravity via Long-term Operations in TianGong-2 Space Lab

De-Sheng Lü,[1,2,*] Wei Ren,[1,2,*] Yuan Sun,[1,†] Tang Li,[1,2] Qiu-Zhi Qu,[1,2] Bin Wang,[1,2] Lin Li,[1,2] Jian-Bo Zhao,[1,2] Xin Zhao,[1,2] Jing-Wei Ji,[1,2] Mei-Feng Ye,[1,2] Jing-Feng Xiang,[1,2] Wei-Biao Chen,[2] Yu-Zhu Wang,[1] and Liang Liu[1,2,‡]

[1] *CAS Key Laboratory of Quantum Optics and Center of Cold Atom Physics, Shanghai Institute of Optics and Fine Mechanics, Chinese Academy of Sciences, Shanghai 201800, China*

[2] *Aerospace Laser Engineering Department, Shanghai Institute of Optics and Fine Mechanics, Chinese Academy of Sciences, Shanghai 201800, China*

(Dated: August 16, 2022)

This supplemental material is organized as the following. (I) More details about the optical system, time-of-flight measurement and in-orbit tests of the experiment. (II) More discussions about the influence of gravity on the polarization gradient cooling (PGC) process.

I. MORE DETAILS OF THE EXPERIMENT

Optical system

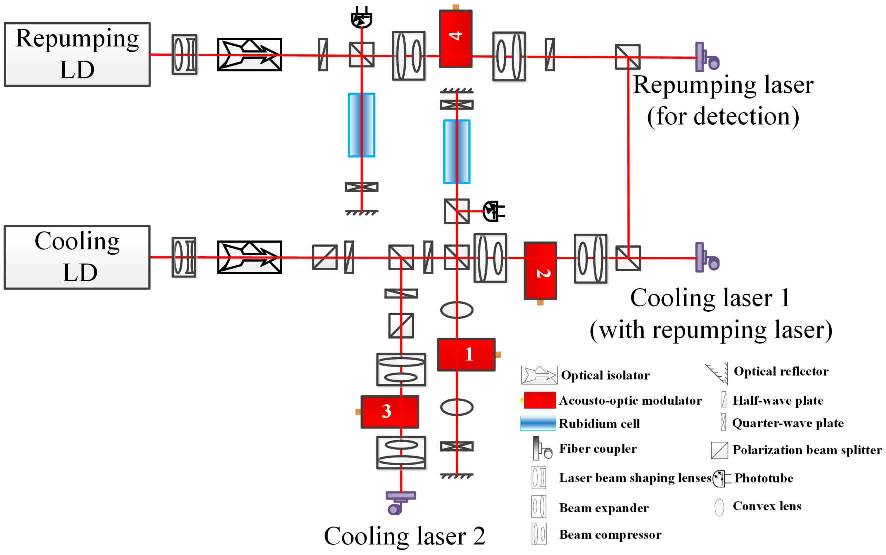

Supplementary Figure S**1**: Simplified schematics of the optical system for the in-orbit laser cooling experiment.

The details of the optical source employed in the in-orbit laser cooling experiments are shown in Fig. S1. The laser sources are the distributed Bragg reflector (DBR) diodes. Each laser diode (LD) is followed by a 40 dB isolator to eliminate the influence of feedback from the surfaces of the downstream optical components. The frequency of cooling light LD is locked to the crossover resonance of the transition $5S_{1/2}, F = 1 \leftrightarrow 5P_{3/2}, F = 2, 3$ of the ^{87}Rb D2 line with the help of an acousto-optic modulator (AOM), by the saturated absorption spectroscopy (SAS) technique. The frequency stabilization process is automated and managed by a micro-controller unit with dedicated software. The laser beam from cooling light LD is divided into different fibres and then serves as cooling laser 1 and cooling laser 2. The frequency of each beam is shifted by an AOM before the fiber coupler (AOM 2 and AOM 3 in Fig. S1).

* These authors contributed equally to the work
† yuansun@siom.ac.cn
‡ liang.liu@siom.ac.cn

Repumping light LD is frequency-locked to the crossover resonance of the transition $5S_{1/2}, F = 1 \leftrightarrow 5P_{3/2}, F = 1, 2$ of the ^{87}Rb D2 line. This beam pass through AOM 4 and is mixed with cooling laser 1 for the subsequent laser cooling process. All the beams from the output of the LDs are collimated and shaped by shaping lenses. Before being injected into the AOMs, the laser beams are compressed by a lens system to enhance the transmission. After passing through AOM followed by a beam expander, the laser beams recover their initial size. In addition, double optical wedges are employed to adjust the coupling efficiency before the fiber coupler. All the optical components are fixed by glue, in order to improve the thermal stability.

Extra details of time-of-flight measurement

The number of cold atoms N can be deduced from the TOF signal according to the following formula:

$$N = \frac{\int U_{PD}(t)dt}{\gamma_p \cdot \Delta t \cdot \alpha \cdot \rho_{PD} \cdot G \cdot E_{\mathrm{Rb}}}, \tag{1}$$

where γ_p, Δt, α, U_{PD}, ρ_{PD} and G represent the scattering rate of the exited level, the time duration of atoms crossing the probe beam, the collection efficiency of fluorescence, the TOF signal line shape, the quantum efficiency of photodiode and the gain of current amplifier, respectively. $E_{\mathrm{Rb}} \approx 2.54 \times 10^{-19}$ J is the energy of ^{87}Rb D2 transition. One set of typical experimental parameter values is: $\gamma_p = 2\pi \times 6.07$ MHz, $\alpha = 0.02$, $\rho_{PD} = 0.5$ A/W, $G = 1.1 \times 10^9$ V/A.

Extra details of in-orbit tests

For Doppler cooling, the maximum damping force takes place when the detuning $\delta = -\gamma/2 \approx -3$ MHz for ^{87}Rb, and the same condition is true for the trapping force of MOT [1]. In order to capture more atoms, however, a larger detuning is necessary to increase the capture velocity. The maximum number of trapped atoms is subject to many factors including the loss rate of cold atoms, the damping force, the trapping potential and the loading time. In the main text, we have already demonstrated results of the relation between the number of loaded cold atoms and the loading time. Here we examine the relation between the number of loaded cold atoms and the cooling laser intensity, and a typical result from the in-orbit MOT tests is shown in Fig. S2. We observe that the number of cold atoms increases linearly with the light intensity, which agrees with the expectations.

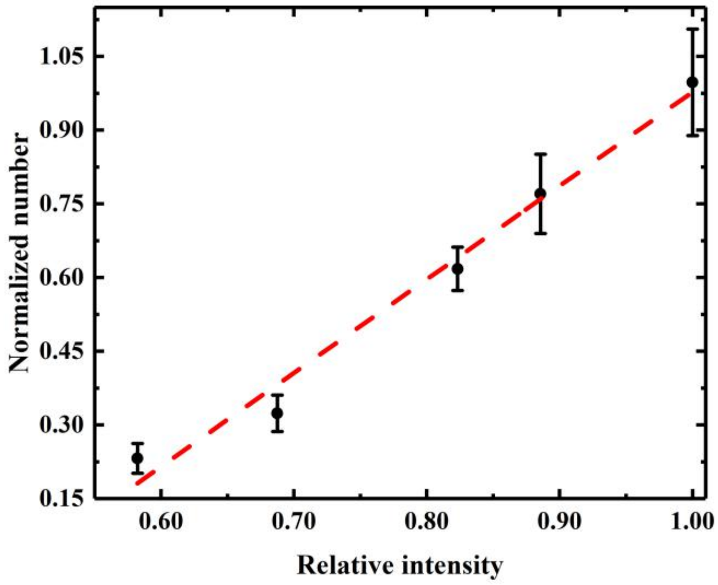

Supplementary Figure S**2**: The relation between cold atom number and the laser intensity.

The magnetic shielding is a crucial factor to ensure the in-orbit performance of our system. It contains both functionalities of passive shielding and active compensation, whose concepts of design have been discussed in Ref. [2].

We have thoroughly examined the magnetic field shielding performance of the apparatus on ground before launch. Under influence of the Earth's magnetic field, the residual magnetic field in the center area is less than 1 nT and the region of uniform magnetic field extends more than 35 cm along the longitudinal direction.

II. THEORETICAL DISCUSSION OF THE GRAVITY'S INFLUENCE ON COLD ATOM TEMPERATURE

Without loss of generality, an atom subject to a generic laser cooling process can be described by the Fokker-Planck equation as:

$$\frac{\partial}{\partial t}W(p,t) = -\frac{\partial\big(F(p,t)W(p,t)\big)}{\partial p} + \frac{\partial^2\big(D(p,t)W(p,t)\big)}{\partial p^2}, \tag{2}$$

where the distribution of atoms' momenta is described by the time-dependent function $W(p,t)$, $F(p,t)$ represents the laser force and $D(p,t)$ is the diffusion coefficient. With respect to generic cooling mechanisms such as Doppler cooling or PGC [3, 4], around a specific value of atom velocity v, we have $F(v) = -\beta v$ and $D(p,t) = D_0$ which is a constant independent of the atomic velocities. The temperature of cold atoms can then be deduced by $k_B T = D_0/\beta$.

For the ideal PGC process in 1D, the force profile can be empirically described as [4]:

$$f(v) = -\zeta\frac{v}{1 + v^2/v_c^2}, \tag{3}$$

whose maximum value occurs at $v = v_c$. With such a description, the force profile is subject to the two constants ζ and v_c, where usually v_c is on the order of a few $0.01\gamma/k$, and the maximum force magnitude ζv_c is usually on the order of $10^{-3}\hbar k\gamma$. In such a formalism, the local damping coefficient is:

$$\beta(v) = -\frac{d}{dv}f(v) = -\zeta\frac{1}{1 + v^2/v_c^2} - 2\zeta\frac{v^2}{v_c^2}\cdot\frac{1}{\big(1 + v^2/v_c^2\big)^2}, \tag{4}$$

where we observe that the magnitude of $\beta(v)$ is largest when $v = 0$, and decreases as the magnitude of v increases. If we consider a simplified situation of only the PGC process taking place, the cold atom temperature can be deduced according to the relation between temperature T and damping coefficient β given by the Fokker-Planck equation. On the other hand, under the presence of gravity, the cold atoms will need to travel at a non-zero velocity v_g to gain a net optical force to balance the gravity. In that way, the cooling force around the central velocity, namely the behavior of β around v_g, becomes less effective under the framework of PGC.

To further investigate the role of gravity in the typical PGC process and check whether the qualitative interpretation makes sense for our in-orbit experimental result, we have carried out a numerical simulation via the method of Monte-Carlo Wave Function (MCWF) [5, 6], also known as the quantum jump approach.

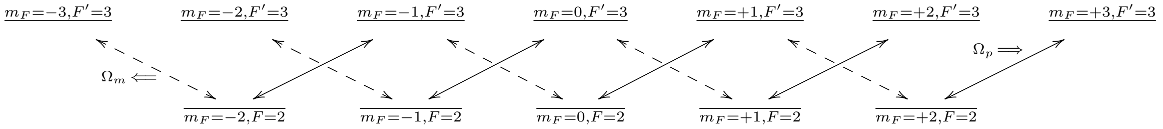

Supplementary Figure S**3**: The linkage structure of our model to numerically simulate the PGC process, with polarization degrees of freedom included. The counter-propagating cooling lasers form a 1D polarization gradient of the ↺⊥↻ configuration.

To reasonably simulate the actual experiment, we consider a model of 12-state system corresponding to the $F = 2 \leftrightarrow F' = 3$ transition ^{87}Rb's D2 line. As shown in Fig. S3, the excited level consists of seven states, which can be recognized as the level of $j = 3$; the ground level consists of five states, which can be recognized as $j = 1, m_j = -1, 0, 1$. We consider a 1D geometry of laser cooling, where one laser travels from negative direction to positive direction with Rabi frequency Ω_p and the other laser travels from positive direction to negative direction with Rabi frequency Ω_m. Both lasers are of the same frequency. These two lasers are assumed to be circularly polarized in the opposite way, such that the Ω_p laser drives the transition of $|m, F = 2\rangle \leftrightarrow |m + 1, F' = 3\rangle$ while the Ω_m laser drives the transition

of $|m, F = 2\rangle \leftrightarrow |m-1, F' = 3\rangle$. We further postulate that these 12 states form a closed system, such that the repumping laser can be neglected for the moment.

We follow a semi-classical approach where the internal degrees of freedom are handled quantum mechanically and the kinematic motion is handled classically. Without decay, the system is described by the atomic wave function of the effective 12-state system and the motion of the atom in terms of (x, v). Under the rotating wave approximation, the time evolution of the wave function can be expressed in terms of ordinary differential equations. For the wave function, we use the notation of $C_{l,m}$, with $l = g, e$ representing the ground level of $F = 2$ and excited level $F' = 3$ respectively, and m standing for m_F. The detuning is defined as $\delta = \omega_{\text{laser}} - \omega_{\text{atom}}$. The Rabi frequency is defined with respect to the strongest transition of $|m_F = 2, F = 2\rangle \leftrightarrow |m_F = 3, F' = 3\rangle$, and the CG coefficients are already included into these equations. Then, the ordinary equation system can be given as:

$$i\frac{d}{dt}\begin{bmatrix} C_{g,-1} \\ C_{g,+1} \\ C_{e,-2} \\ C_{e,0} \\ C_{e,+2} \end{bmatrix} = \begin{bmatrix} -kv & 0 & \frac{1}{\sqrt{6}}\Omega_m(x) & \frac{1}{2\sqrt{5}}\Omega_p(x) & 0 \\ 0 & kv & 0 & \frac{1}{2\sqrt{5}}\Omega_m(x) & \frac{1}{\sqrt{6}}\Omega_p(x) \\ \frac{1}{\sqrt{6}}\Omega_m^*(x) & 0 & -\delta - 2kv & 0 & 0 \\ \frac{1}{2\sqrt{5}}\Omega_p^*(x) & \frac{1}{2\sqrt{5}}\Omega_m^*(x) & 0 & -\delta & 0 \\ 0 & \frac{1}{\sqrt{6}}\Omega_p^*(x) & 0 & 0 & -\delta + 2kv \end{bmatrix} \cdot \begin{bmatrix} C_{g,-1} \\ C_{g,+1} \\ C_{e,-2} \\ C_{e,0} \\ C_{e,+2} \end{bmatrix}; \tag{5}$$

$$i\frac{d}{dt}\begin{bmatrix} C_{g,-2} \\ C_{g,0} \\ C_{g,+2} \\ C_{e,-3} \\ C_{e,-1} \\ C_{e,+1} \\ C_{e,+3} \end{bmatrix} = \begin{bmatrix} -2kv & 0 & 0 & \frac{1}{2}\Omega_m & \frac{1}{2\sqrt{15}}\Omega_p & 0 & 0 \\ 0 & 0 & 0 & 0 & \frac{1}{\sqrt{10}}\Omega_m & \frac{1}{\sqrt{10}}\Omega_p & 0 \\ 0 & 0 & 2kv & 0 & 0 & \frac{1}{2\sqrt{15}}\Omega_m & \frac{1}{2}\Omega_p \\ \frac{1}{2}\Omega_m^* & 0 & 0 & -\delta - 3kv & 0 & 0 & 0 \\ \frac{1}{2\sqrt{15}}\Omega_p^* & \frac{1}{\sqrt{10}}\Omega_m^* & 0 & 0 & -\delta - kv & 0 & 0 \\ 0 & \frac{1}{\sqrt{10}}\Omega_p^* & \frac{1}{2\sqrt{15}}\Omega_m^* & 0 & 0 & -\delta + kv & 0 \\ 0 & 0 & \frac{1}{2}\Omega_p^* & 0 & 0 & 0 & -\delta + 3kv \end{bmatrix} \cdot \begin{bmatrix} C_{g,-2} \\ C_{g,0} \\ C_{g,+2} \\ C_{e,-3} \\ C_{e,-1} \\ C_{e,+1} \\ C_{e,+3} \end{bmatrix}. \tag{6}$$

The spontaneous emission is treated as quantum jumps with the help of the pseudo random number generators, according to the framework of MCWF method. The destination of spontaneous emission is set as the randomized linear superposition of possible states in the ground level of $F = 2$, in order to better emulate the optical pumping effects. Then the acceleration received by the atom is treated classically as the photon recoil whenever the spontaneous emission takes place. We have also included the phase noise of the cooling lasers into the simulation, as we set its line width as 100 kHz. From the numerical simulation we observe that the PGC effect of the $\circlearrowleft \perp \circlearrowright$ configuration is indeed caused by the asymmetry of scattering when the atom is traveling at relatively low velocities.

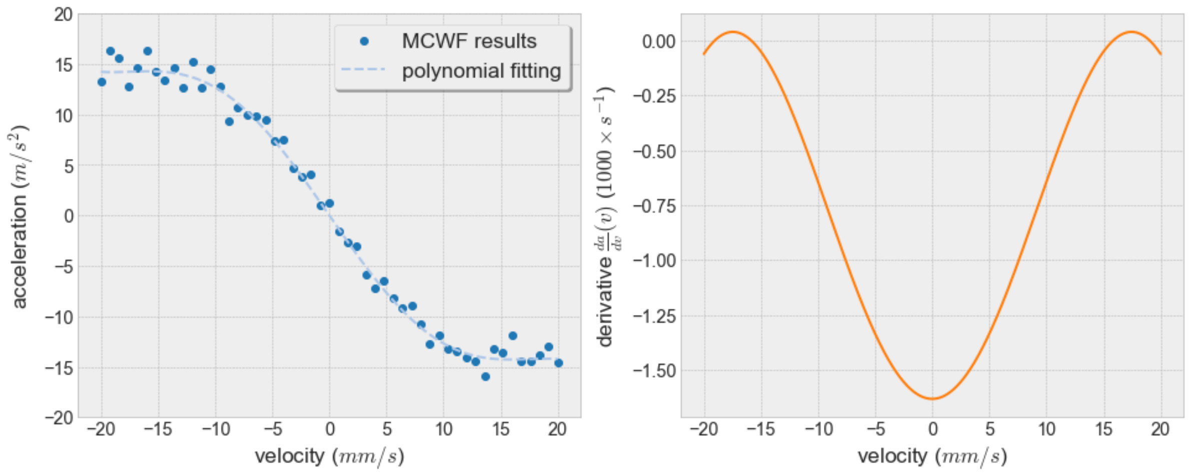

Supplementary Figure S**4**: Acceleration vs. velocity of the PGC process, according to the numerical simulations in zero magnetic field. Each acceleration value is computed from averaging 100,000 MCWF trajectories. A fitting to the polynomial function in the format of $ax + bx^3 + cx^5 + dx^7$ is also shown, and the derivative of such a polynomial function is also plotted in the figure on the right.

With this MCWF numerical simulation, we have computed the force vs. velocity profile of the PGC process. In particular, we set the parameters as the typical values employed in the experiment with ^{87}Rb D2 transition, with $\Omega_p = \Omega_m = 2\pi \times 8$ MHz, $\delta = 2\pi \times -42$ MHz, the decay rate $\gamma_e = 2\pi \times 6.07$ MHz, the recoil velocity $v_r = 5.88 \times 10^{-3}$ m/s, and the optical wavelength of transition $\lambda = 780.2$ nm. Since here we are purely computing the force profile, the heating effects caused by the random walk of recoil events are not included in the calculation. The result is shown in Fig. S4. Without the presence of gravity, the PGC force profile is nicely performing for small velocities and symmetric around zero velocity, as anticipated. On the other hand, when the gravity does exist as an extra acceleration $g = 9.8m/s^2$ on top of Fig. S4, the atom needs to keep a substantial non-zero velocity v_g to maintain a net zero acceleration, with less cooling efficiency as can be observed from the numerical results.

On average, the measured cold atom temperature is $T_0 = 3.3$ μK in microgravity and $T_g = 7.3$ μK on ground (see main text for the details of experimental results), with the ratio $T_g/T_0 \approx 2.2$. The numerical simulation result of Fig. S4 is within a reasonable range with the experimental result. Although the numerical simulation is carried out via a effectively simplified model, but its outcome provides straightforward interpretations into the underlying physics.

We note that, in fact, it is the gravity which reduces the efficiency of laser cooling and trapping on ground. The increased efficiency of laser cooling in microgravity implies that in-orbit environment offers a better place to work on cold atom physics. Last but not least, we note that the practical situation is more complicated than the above simplified discussions, and the PGC process is much more complicated than the simplified numerical model or the empirical formula. In other words, the influence of the gravity on the ultimate temperature of the typical laser cooling processes is a relatively complicated issue worth further studies.

[1] Metcalf HJ and van der Straten P. *Laser Cooling and Trapping*, (Springer-Verlag, New York1999).

[2] Li L, Ji J, Ren W *et al.* Automatic compensation of magnetic field for a rubidium space cold atom clock. *Chinese Physics B* 2016; **25**: 073201.

[3] Shahriar MS, Hemmer PR, Prentiss MG *et al.* Continuous polarization-gradient precooling-assisted velocity-selective coherent population trapping. *Phys. Rev. A* 1993; **48**: R4035–R4038.

[4] Devlin JA and Tarbutt MR. Three-dimensional doppler, polarization-gradient, and magneto-optical forces for atoms and molecules with dark states. *New Journal of Physics* 2016; **18**: 123017.

[5] Dalibard J, Castin Y and Mølmer K. Wave-function approach to dissipative processes in quantum optics. *Phys. Rev. Lett.* 1992; **68**: 580–583.

[6] Plenio MB and Knight PL. The quantum-jump approach to dissipative dynamics in quantum optics. *Rev. Mod. Phys.* 1998; **70**: 101–144.
